# The role of hematological parameters in asymptomatic and non-severe cases of Omicron variant infection

**DOI:** 10.1186/s12985-024-02414-x

**Published:** 2024-06-24

**Authors:** Suqin Ben, Fengying Gao, Ziheng Xu, Rulin Zhang, Xingyi Zhang, Ning Wang, Min Zhang, Lili Hou

**Affiliations:** 1 Department of Respiratory and Critical Care Medicine, Shanghai General Hospital, Shanghai Jiao Tong University School of Medicine, 100 HainingRoad, Hongkou District, Shanghai, 200080 China; 2 Department of Infectious Diseases, Shanghai General Hospital, Shanghai Jiao Tong University School of Medicine, Shanghai, 201620 China; 3 Department of Pulmonary Disease, Shanghai Municipal Hospital of Traditional Chinese Medicine, Shanghai University of Traditional Chinese Medicine, Shanghai, 200071 PR China; 4 Department of Laboratory Medicine, Shanghai General Hospital, Shanghai Jiao Tong University School of Medicine, Shanghai, 200080 China; 5 Department of Respiratory and Critical Care Medicine, Jiuquan Branch of Shanghai General Hospital, Gansu, 735099 China

**Keywords:** Omicron, Hematological parameters, Basophiles, SARS-CoV-2

## Abstract

**Background:**

Omicron variants are currently the predominant circulating lineage worldwide and most cases are mild or asymptomatic. The Omicron variant is characterized by high transmissibility and immune evasion. Early identification of Omicron cases in clinical settings is crucial for controlling its spread. Previous studies have indicated that changes in hematological parameters can be used to predict the severity of coronavirus disease 2019 (COVID-19). However, the role of hematological parameters in non-severe and asymptomatic cases remains unknown. This study aimed to investigate the role of hematological parameters in non-severe and asymptomatic Omicron variant infections.

**Methods:**

Hematological parameters and results were analyzed and compared in symptomatic (*n* = 356) and asymptomatic (*n* = 171) groups respectively, and between these two groups with positive COVID-19 tests. The utility of hematological parameters for predicting positive COVID-19 tests was analyzed using receiver operating characteristic curves.

**Results:**

Individuals with non-severe cases exhibited decreased levels of platelets, lymphocytes, eosinophils, basophils, lymphocytes (%), eosinophils (%), and basophils (%), while exhibiting elevated counts of monocytes, neutrophils (%), monocytes (%), neutrophil-to-lymphocyte ratio, platelet-to-lymphocyte ratio (PLR), and C-reactive protein (CRP) when compared to suspected cases or asymptomatic carriers. In asymptomatic patients, positive carriers had lower leukocyte, neutrophil, and lymphocyte counts but higher monocyte, monocyte (%), PLR, and CRP levels than negative carriers. Basophil counts combined with lymphocytes or the PLR demonstrated a more significant predictive value in screening non-severe cases earlier compared to other parameters. The combined assessment of the monocyte (%) and the PLR had the highest area under the curve for diagnosing asymptomatic carriers.

**Conclusions:**

Circulating basophils, alone or in combination with other hematological parameters, may be used as efficient biomarkers for early screening of non-severe Omicron cases.

## Background

Severe acute respiratory syndrome coronavirus 2 (SARS-CoV-2) is the causative pathogen of the coronavirus disease 2019 (COVID-19) pandemic. The virus continues to evolve and mutate rapidly. Omicron variants have predominated worldwide since they were first identified in South Africa in November 2021. Over time, several sub-variants emerged, namely, BA.1, BA.2, BA.3, BA.4, BA.5, XBB, XBD, and XBF. Since their emergence, Omicron variants have spread rapidly to Europe, Asia, Africa, and the United States, generating a new wave called the Omicron wave [1–5]. More than 130 million cases, including 500,000 deaths, have been reported globally, representing a 44% increase in the average number of COVID-19 cases [6].

Omicron variants are more transmissible and less susceptible to vaccines compared to other strains [2, 7]. The symptoms of Omicron infection appear to be less dangerous compared to the acute symptomatic presentations observed with previous SARS-CoV-2 strains [8, 9]. It has been reported that 91% of cases are asymptomatic [10]. Omicron infections are associated with a small proportion of severe cases [2, 8, 11]. Nevertheless, previous studies have reported that unvaccinated individuals, especially older adults with underlying health conditions, are at higher risk of developing severe or critical illness [2, 12]. In China, approximately 49 million people aged > 60 years have not yet been vaccinated. Moreover, many of these patients have preexisting comorbidities. The COVID-19 outbreak has overwhelmed healthcare systems and caused massive economic losses in China. Therefore, early identification of patients infected with Omicron, in combination with epidemiological investigations, is crucial. However, rapidly screening potential cases among close contacts and devising appropriate treatment plans immediately in clinics pose great challenges for physicians.

SARS-CoV-2 rapid antigen diagnostic tests (Ag-RDTs) are used worldwide for the detection of the Omicron variant to prevent the spread of COVID-19 due to its convenient and rapid turnaround time [13]. Nonetheless, the gold standard for confirming COVID-19 diagnosis remains the real-time reverse transcriptase-polymerase chain reaction (RT-PCR) assay [14]. However, as the prevalence of Omicron transitions from a COVID-19 wave into a ‘wavelet’ era [15], large-scale nucleic acid testing using these two methods is no longer a routine screening method. Therefore, discovering simple and effective measures to screen potential cases earlier could provide great value in preventing the spread of Omicron variants. Routine complete blood count (CBC) tests are a conventional method for screening infectious diseases in clinical settings. Given their importance in assessing overall health during hospital visits, these tests might offer a unique opportunity to rapidly screen for potential Omicron cases.

Hematological parameters, such as the neutrophil-to-lymphocyte ratio (NLR), platelet-to-lymphocyte ratio (PLR), and eosinophil count (EOS), are useful for predicting COVID-19 diagnosis and evaluating its severity according to early studies [16, 17]. Basophils (BAS) account for less than 1% of blood-circulating leukocytes. As the rarest granulocytes, BAS can induce Th2 cell differentiation, and their depletion leads to greater susceptibility to infection [18]. Several studies have indicated a decrease in BAS in patients with COVID-19 [16, 19–27], and lower BAS counts may predict poorer patient outcomes [25]. However, previous studies have mainly focused on patients with common and severe manifestations of COVID-19 and have not evaluated the role of hematological parameters in mild and asymptomatic patients. Most importantly, whether these parameters could be used to predict Omicron variant infection in mildly symptomatic or asymptomatic participants remains unclear, considering the different immune responses to different SARS-CoV-2 variants.

This study aimed to explore the alterations and functions of hematological parameters in non-severe patients, particular in those with mild infections and asymptomatic carriers. Additionally, it sought to assess the value of BAS, either independently or in combination with other hematological parameters, for predicting the diagnosis of omicron variant infection in mild and asymptomatic patients. This will contribute to distinguishing between mild cases and asymptomatic carriers at an early stage among close contacts, which will be helpful in treating Omicron variant infections earlier.

## Methods

### Study population and study design

This retrospective observational study recruited 1169 adult patients who were in close contact with definitively diagnosed COVID-19 patients. These patients visited the fever clinics, the Pulmonary Outpatient Clinic, or special isolation wards (used to isolate the suspected cases and the Omicron variant-confirmed cases) of Shanghai General Hospital affiliated with Shanghai Jiao Tong University between January 2022 and May 2022. The patients underwent an epidemiological investigation and were followed up via telephone. RT-PCR assays of nasal and pharyngeal swab specimens were performed for all cases. Patients with RT-PCR Ct values < 35 were considered positive and confirmed to be infected with Omicron variants according to the ninth edition of *the Novel Coronavirus and Pneumonia Diagnosis and Treatment Interim Guidance* Report issued by the National Health Commission of the People’s Republic of China. Additionally, the following clinical types of COVID-19 were also defined in accordance with this *Guidance*.

Among these patients, 803 were symptomatic and 366 were asymptomatic. Symptomatic patients exhibited symptoms such as fever, sore throat or pharyngeal discomfort, hoarseness, nasal congestion or runny nose, sneezing, chills, muscle or body aches, fatigue, cough, sputum production, headache, dizziness, nausea or vomiting, diarrhea, or abdominal discomfort lasting for less than 3 days. Asymptomatic carriers were individuals who had normal findings on high-resolution computed tomography (HRCT) imaging and experienced no symptoms but tested positive for novel coronavirus. Mild cases were symptomatic with positive COVID tests and normal HRCT imaging. Common cases were defined as those presenting symptoms along with typical chest HRCT imaging changes, including peripheral pulmonary multilobular plaques/interstitial lesions, bilateral multiple lobular and subsegmental areas of ground-glass opacities, or consolidation [17]. Mild cases and common cases were recognized as non-severe cases in this study. Severe cases was characterized by the following criteria: respiratory frequency ≥ 30 breaths per min, SpO_2_ < 94% on room air at sea level, a ratio of the arterial partial pressure of oxygen to fraction of inspired oxygen (PaO_2_/FiO_2_) ≤ 300, or lung infiltrates > 50% within a 48 h period. Suspected cases included symptomatic patients with negative COVID tests.

The criteria for patient inclusion were as follows: (1) patients aged 16–79 years; (2) epidemiological investigations indicating that participants were close contacts of patients with COVID-19; (3) patients with negative serum influenza A or B IgM; and (4) blood routine tests (Mindray, BC-5390CRP, China), CRP assays (Mindray, BC-5390CRP, China), RT-PCR for detecting SARS-CoV-2 nucleic acid qualitatively using nasopharyngeal swabs, and chest HRCT (slice thickness, 0.625 mm, GE medical system) performed during clinic visits or in the special isolation ward. All tests were performed within 5 days of patients being identified as close contacts of COVID-19 cases.

The exclusion criteria comprised patients with active pulmonary tuberculosis, bronchiectasis, neoplastic disease, asthma, asthma-chronic obstructive pulmonary disease (COPD) overlap syndrome (ACOS), interstitial lung disease, rhinitis, autoimmune diseases, significant food allergies and receiving immunotherapy, severe unstable COPD, or any exacerbation of COPD during the previous 6 months. Additionally, pregnant women were excluded, as were patients with underlying diseases such as acute intestinal obstruction, acute gastroenteritis, or acute-appendicitis, which might affect blood parameters counts. Severe cases of COVID-19 were also excluded from this study.

**Fig. 1:**
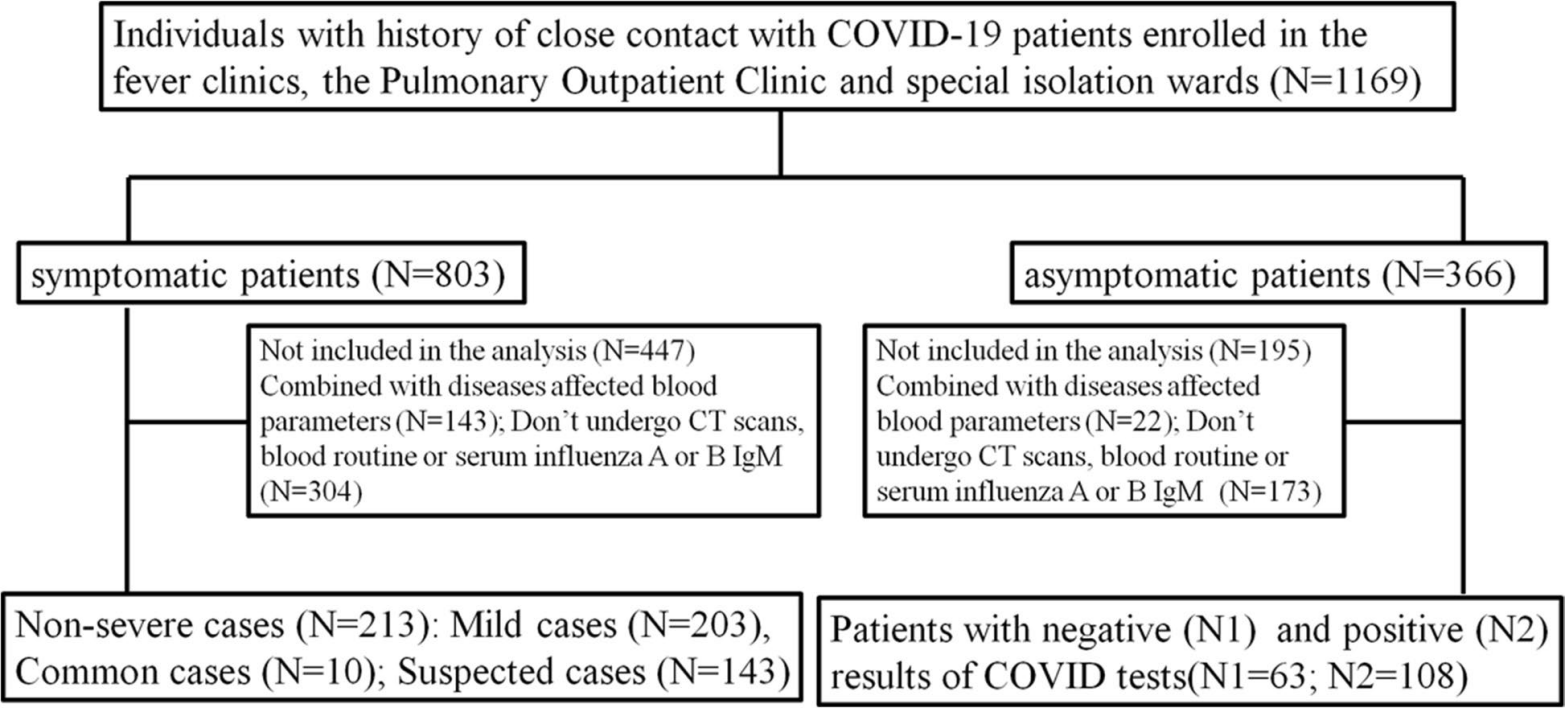
Flow diagram for participants through the study. The data were collected between January 1, 2022 and May 31, 2022. COVID-19, Coronavirus disease 2019; CT, computerized tomography

Among the symptomatic patients included, 203 were classified as mild cases, while 10 were categorized as common cases. Additionally, there were 143 suspected cases (Fig. 1). Among the 171 asymptomatic patients, 108 tested positive, while 63 tested negative (Fig. 1).

Demographic data and laboratory values were extracted from electronic medical records and patient files. The Ethics Committee of the Institutional Review Board at Shanghai General Hospital (no. 2024KS189) approved this study.The study was conducted in accordance with the relevant guidelines and regulations/ethical principles of the Declaration of Helsinki. A waiver of informed consent was obtained from the study participants.

### Outcomes

The primary outcome was to evaluate the predictive value of the BAS count, either alone or in combination with other peripheral blood parameters, for the diagnosis of mild cases among close contacts. The secondary outcome was to assess the diagnostic potential of the combined assessment of the PLR and monocyte (MO) (%) in accurately identifying asymptomatic carriers of the SARS-CoV-2 virus.

### Statistical analysis

Data were analyzed for normality of distribution using the Kolmogorov–Smirnov test. Normally distributed data were presented as the mean ± standard deviation. Non-normally distributed data were expressed as medians and interquartile ranges. Independent samples were compared using Student’s *t*-test (two-tailed) or the Mann–Whitney U test. Count data are presented as percentages and between-group comparisons were performed using the chi-square test (χ^2^).

Nucleic acid testing is considered the gold standard for diagnosing positive cases in patients with and without symptoms. Logistic regression was applied to determine the impact of continuous test variables on dichotomous state variables. Univariate logistic regression was used to determine the impact of the variables of interest. Subsequently, the variables of interest were normalized as $x=\frac{\text{variables}-mean}{\text{standard deviation}}$ and multiple logistic regression was performed to ascertain whether the model could be improved. The predictive values of single or combined normalized variables were calculated by constructing receiver operating characteristic (ROC) curves and measuring the area under the curves (AUCs) [28]. In the ROC plot, sensitivity was plotted against the false positive rate (100-specificity), and the cut-off value was determined based on Youden’s index.

Statistical analyses were performed using SPSS version 22.0 (IBM Corp., Armonk, NY, USA). ROC curve construction and AUC comparisons were performed using MediCalc 19.0.4 software. AUCs were compared using the χ^2^ test based on the method of Hanley and McNeil. Statistical significance was set at *P* < 0.05.

## Results

### Baseline characteristics of the positive cases

A total of 1169 patients with a history of close contact with COVID-19 patients via epidemiological investigation were enrolled. Among these patients, 803 were symptomatic and 366 were asymptomatic. Finally, 356 symptomatic participants and 171 asymptomatic individuals were included after applying the inclusion and exclusion criteria (Fig. 1).

A total of 356 (67.55%) patients exhibited fever, sore throat or pharyngeal discomfort, hoarseness, nasal congestion or runny nose, sneezing, chills, muscle ache, fatigue, cough, sputum production, headache, dizziness, and chest tightness (Table 1). Of these, 213 (59.83%) patients tested positive for the Omicron variant, and 136 of these individuals showed combined symptoms. Among the 203 mild cases, 185 (91.13%) were vaccinated (Table 2). Additionally, 131 out of 139 (94.24%) suspected cases were vaccinated as well (Table 2). The other 4 suspected cases did not report their vaccination status. Of the 203 mild cases, three (1.48%) had a positive re-test of viral RNA. No significant difference was observed in the median interval from the onset of fever to hospital visits between the symptomatic groups (data not shown). In addition to fever and nasopharyngeal symptoms, the most common symptoms were coughing, fatigue, headache, sputum production, dizziness, nausea, and vomiting (Table 1). Muscle or body aches were more common in non-severe cases than in suspected cases (Table 1).

**Table 1:** Symptoms comparisons of suspected cases and non-severe cases

Characteristic variables	Suspected cases	Non-severe cases	*P* value
	n_1_ = 143	n_2_ = 213	
Fever	52 (36.36%)	100 (46.95%)	**0.048**^Ψ^
Sore throat/ Pharyngeal discomfort	36 (25.17%)	46 (21.60%)	0.432^Ψ^
Hoarseness	2 (1.40%)	2 (0.94%)	1.000^$$^
Nasal congestion/runny nose	17 (11.89%)	16 (7.51%)	0.163^Ψ^
Sneeze	2 (1.40%)	0 (0.00%)	0.161 ^$$^
Chills	3 (2.10%)	6 (2.82%)	0.937^$^
Muscle/Body ache	9(6.29%)	28 (13.15%)	**0.038**^Ψ^
Hyperhidrosis	0 (0.00%)	1 (0.47%)	1.000^$$^
Tiredness	12 (8.39%)	18 (8.45%)	0.984^Ψ^
Cough	37 (25.87%)	39 (18.31%)	0.088^Ψ^
Sputum production	13(9.10%)	12 (5.63%)	0.211^Ψ^
Headache	30 (20.98%)	22 (10.33%)	**0.005**^Ψ^
Dizziness	4 (2.80%)	10 (4.69%)	0.366^Ψ^
Hypogeusesthesia	0 (0.00%)	1 (0.47%)	1.000^$$^
Anorexia	0 (0.00%)	1 (0.47%)	1.000^$$^
Nausea or vomiting	7 (4.90%)	14 (6.57%)	0.510^Ψ^
Diarrhea or Abdominal discomfort	2 (1.40%)	7 (3.18%)	0.442^$^
Chest pain	1 (0.70%)	1 (0.45%)	1.000^$$^
Chest tightness	3 (2.10%)	4 (3.29%)	1.000^$$^
Chest CT (pneumonia, %)	5 (3.50%)	10 (4.69%)	0.581^Ψ^

Bold font indicates statistical significance

*p* values comparing the group of COVID-19 cases and other groups are from χ^2^ test

^Ψ^Pearson’s χ^2^

^$$^Fisher’s Exact test

^$^Continuity correction

**Table 2:** Demographic data and baseline hematological parameters of patients with symptoms in suspected cases and non-severe cases

Characteristic variables	Suspected cases	Non-severe cases	*P* value
	n_1_ = 143	n_2_ = 213	
Male (n /%)	73 (51.05%)	111 (52.11%)	0.844
Age, Years^a^	32.00 (25.00, 40.00)	32.00 (27.00, 43.00)	0.294
BMI (kg/m^2^)	22.83 ± 2.06	23.02 ± 3.09	0.492
Smoking history			0.084
Never smoked	126 (88.1%)	169 (82.9%)	
Former smoker	3 (2.1%)	6 (2.5%)	
Current smoker	14 (9.8%)	38 (14.6%)	
Vaccination^c^	131 (94.24%)	185 (91.13%)	0.286
**Complications**			
Emphysema	0 (0)	2 (0.94%)	0.518^$$^
Coronary heart disease	0 (0)	2 (0.94%)	0.518^$$^
Heart failure	0 (0)	2 (0.94%)	0.518^$$^
Hepatitis B infection	0 (0)	1 (0.47%)	1.000^$$^
Diabetes	0 (0)	1 (0.47%)	1.000^$$^
Hypertension	0 (0)	2 (0.94%)	0.518^$$^
Depression	1 (0.70%)	1 (0.47%)	1.000^$$^
**Blood parameters (Reference Value)**			
RBCs (3.68–5.13 × 10^12^/L)^a^	4.64 (4.34,5.04)	4.81 (4.45,5.21)	**0.023**
Haemoglobin (113–151 g/L)^b^	142.94 ± 14.99	144.85 ± 18.00	0.280
Haematocrit (33.5%-45.0%)^b^	42.64 ± 4.04	43.01 ± 4.75	0.426
Platelets (85–303 × 10^9^/L)^a^	220.0 (178.0,266.0)	190.0(160.0,220.0)	** < 0.001**
WBCs (4.0–10.0 × 10^9^/L)^a^	7.67 (5.63,9.48)	6.15 (4.84,7.65)	** < 0.001**
< 4	10 (6.99%)	21 (9.86%)	**-**
4–10	104 (72.73%)	175 (82.16%)	**-**
> 10	29 (20.28%)	17 (7.98%)	**-**
Neutrophils (2.0–7.0 × 10^9^/L)^a^	4.90 (3.33,6.79)	4.41 (3.29,5.81)	**0.041**
< 2	9 (6.29%)	16 (7.51%)	**-**
2–7	101 (70.63%)	164 (77.00%)	**-**
> 7	33 (23.08%)	33 (15.49%)	
Lymphocytes(0.8–4.0 × 10^9^/L)^a^	1.68 (1.23,2.13)	1.00 (0.61,1.48)	** < 0.001**
< 0.8	6 (4.20%)	71 (33.33%)	**-**
0.8–4.0	137 (95.80%)	142 (66.67%)	**-**
Eosinophils (0.02–0.5 × 10^9^/L)^a^	0.08 (0.03,0.16)	0.02 (0.01,0.06)	** < 0.001**
< 0.02	20 (13.99%)	97 (45.54%)	**-**
≥ 0.02	123 (86.01%)	116 (54.46%)	**-**
Basophiles (0.00–1.00 × 10^9^/L)^a^	0.02 (0.01,0.03)	0.01 (0.00,0.01)	** < 0.001**
Monocytes (0.12–1 × 10^9^/L)^a^	0.45 (0.33,0.58)	0.51 (0.37,0.65)	**0.026**
Neutrophils (40–70%)^a^	67.90 (59.10,75.20)	72.90 (64.00,82.10)	** < 0.001**
Lymphocytes (20–40%)^a^	24.30 (16.00,32.10)	17.40 (10.20,25.90)	** < 0.001**
Monocytes (3–10%)^a^	5.80 (4.60,8.20)	8.10 (6.25,11.40)	** < 0.001**
Eosinophils (0.5–5%)^a^	1.00 (0.40,2.10)	0.30 (0.10,0.90)	** < 0.001**
Basophiles (0–1%)^a^	0.30 (0.20,0.40)	0.10 (0.10,0.20)	** < 0.001**
NLR^a^	2.77 (1.85,4.29)	4.22 (2.48,7.82)	** < 0.001**
PLR^a^	138.13 (106.72,163.95)	193.13(125.18,312.26)	** < 0.001**
CRP (0-10 mg/L)^a^	4.30(1.30,14.00)	6.15 (3.08,12.85)	**0.031**

Bold font indicates statistical significance

*p* values comparing the group of COVID-19 cases and other groups are from Pearson’s χ^2^ test, Student’s *t* test (2-tailed) or Mann–Whitney U test

*NLR* neutrophil-to-lymphocyte ratio, *PLR* platelet-to-lymphocyte ratio, *RBCs* red blood cells, *WBCs* white blood cells, *CRP* C-reactive protein

^$$^Fisher’s Exact test

^a^median (IQR) values

^b^mean ± SD values

^c^n1 = 139, n2 = 203 mild cases

Based on the COVID test results, there were no significant between-group differences in age or sex. Non-severe cases exhibited lower platelet (PLT), white blood cell (WBC), neutrophil (Neu), lymphocyte (Lym), EOS, BAS, Lym (%), EOS (%), and BAS (%) counts (all *P* < 0.05) (Table 2). Additionally, the red blood cell (RBC), MO, neutrophil (%) and MO (%) counts, as well as the NLR, PLR, and C-reactive protein (CRP) levels were significantly higher in non-severe cases than in suspected cases (all *P* < 0.05) (Table 2).

In total, 171 participants were asymptomatic. Among these, 108 tested positive for the Omicron variant. Of the 91 asymptomatic carriers, 83 (91.21%) were vaccinated (Table 3). Overall, 48 asymptomatic patients with negative COVID test results reported their vaccination status, and 46 individuals (95.83%) were vaccinated (Table 3). Of the 91 asymptomatic carriers, two (2.20%) had positive re-test viral RNA results. Several significant differences were observed, including lower WBC, Neu, and Lym counts, and higher MO and MO (%) counts, as well as higher PLRs and CRP levels in the positive group compared to the negative group (Table 3).

**Table 3:** Demographic data and baseline hematological parameters of asymptomatic patients in negative and positive group

Characteristic variables	Negative cases	Positive cases	*P* value
	n_1_ = 63	n_2_ = 108	
Male (n /%)^a^	34(53.97%)	50 (46.30%)	0.333
Age, Years^a^	35.0 0(27.00, 51.00)	34.00 (25.00, 49.75)	0.429
BMI (kg/m^2^)	22.55 ± 2.88	22.89 ± 2.68	0.431
Smoking History			0.852
Never smoked	52 (82.5%)	89 (84.8%)	
Former smoker	2 (3.2%)	4 (3.8%)	
Current smoker	9 (14.3%)	12 (11.4%)	
Vaccination^c^	46 (95.83%)	83 (91.21%)	0.295
**Complications**			
Emphysema	2 (3.17%)	1 (0.93%)	0.634^$^
Coronary heart disease	1 (1.59%)	3 (2.78%)	1.000^$^
Cerebrovascular disease	2 (3.17%)	0 (0)	0.134^$$^
Hypertension	2 (3.17%)	1 (0.93%)	0.634^$^
Depression	0 (0)	1 (0.93%)	1.000^$$^
**Blood parameters**			
RBCs (3.68–5.13 × 10^12^/L)^b^	4.83 ± 4.85	4.79 ± 0.51	0.601
Haemoglobin (113–151 g/L)^b^	145.43 ± 15.23	144.51 ± 16.08	0.714
Haematocrit (33.5%-45.0%)^b^	43.35 ± 4.24	43.17 ± 4.53	0.803
Platelets (85–303 × 10^9^/L)^a^	225.00 (195.00,267.00)	222.00 (183.00,255.75)	0.382
WBCs (4.0–10.0 × 10^9^/L)^a^	7.35 (6.12,8.31)	6.19 (5.03,9.78)	**0.001**
< 4	1 (1.59%)	9 (8.33%)	
4–10	56 (88.89%)	94 (87.04%)	
> 10	6 (9.52%)	5 (4.63%)	
Neutrophils (2.0–7.0 × 10^9^/L)^a^	4.53 (3.85,6.07)	4.24 (3.23,5.10)	**0.018**
< 2	1 (1.59%)	9 (8.33%)	
2–7	54 (85.71%)	90 (83.33%)	
> 7	8 (12.70%)	9 (8.33%)	
Lymphocytes (0.8–4.0 × 10^9^/L)^a^	1.85 (1.40,2.17)	1.42 (0.98,1.91)	**< 0.001**
< 0.8	1 (1.59%)	15 (13.89%)	
0.8–4.0	62 (98.41%)	93 (86.11%)	
Eosinophils (0.02–0.5 × 10^9^/L)^a^	0.05 (0.03,0.13)	0.06 (0.02,0.11)	0.561
< 0.02	7 (11.11%)	16 (14.81%)	
≥ 0.02	56 (88.89%)	92 (85.19%)	
Monocytes (0.12–1 × 10^9^/L)^a^	0.38(0.29,0.47)	0.43 (0.32,0.58)	**0.028**
Basophils (0.00–1.00 × 10^9^/L)^a^	0.01 (0.01,0.02)	0.01 (0.01,0.02)	0.934
Neutrophils (40–70%)^b^	66.90 ± 9.95	66.26 ± 12.58	0.727
Lym (20–40%)^b^	26.02 ± 8.86	24.43 ± 11.13	0.333
MO (3–10%)^a^	5.40 (3.90,6.30)	6.90 (5.03,9.78)	**< 0.001**
EOS(0.5–5%)^a^	0.90 (0.40,1.90)	1.05 (0.40,1.90)	0.771
BAS(0–1%)^a^	0.20 (0.10,0.30)	0.25 (0.10,0.30)	0.368
NLR^a^	2.52 (1.68,3.83)	2.73 (1.85,4.64)	0.368
PLR^a^	136.57 (101.62,165.66)	155.87 (120.71,224.47)	**0.006**
C-reactive protein (0-10 mg/L)^a^	1.00 (0.50,1.98)	2.75 (1.20,7.68)	**< 0.001**
**Chest CT (pneumonia, %)**	5 (7.94%)	3 (2.78%)	0.244

Bold font indicates statistical significance

*p* values comparing the group of COVID-19 cases and other groups are from χ^2^ test, Student’s *t* test (2-tailed) or Mann–Whitney U test

*Lym* Lymphocytes, *BAS* basophile, *EOS* eosinophils, *MO* Monocytes, *Neu* neutrophils, *NLR* neutrophil-to-lymphocyte ratio, *PLR* platelet-to-lymphocyteratio, *RBCs* red blood cells, *WBCs* white blood cells, *CRP* C-reactive protein

^$^Continuity correction

^$$^Fisher’s Exact test

^a^median (IQR) values

^b^mean ± SD values

^c^n1 = 48, n2 = 91

### Predictive values of single and combined variables for diagnosis of non-severe cases

The predictive values of PLT, lymphocyte, MO, EOS, and BAS counts, lymphocyte (%), MO (%), EOS (%), BAS (%), NLR, and PLR, either alone or in combination, were evaluated using ROC curves. Table 4 presents the sensitivity, specificity, PPV, NPV, and accuracy of each combined variable.

**Table 4:** Predictive values of single and combined variables for non-severe cases (*n* = 356)

Characteristic variables	AUC	Cut off values^*^	Sensitivity (%)	Specificity (%)	PPV (%)	NPV (%)	Accuracy (%)	+ LR	-LR	Variable *P*
**Single variable**										
PLT	0.648	≤ 208	67.14	59.44	71.14	54.84	64.04	1.66	0.55	< 0.001
Normalized Lym	0.766	≤ -0.20	62.91	76.92	80.24	58.20	68.54	2.73	0.48	< 0.001
MO	0.570	> 0.53	44.60	70.63	69.34	46.12	55.06	1.52	0.78	0.024
EOS	0.748	≤ 0.02	60.09	78.32	80.50	56.85	67.42	2.77	0.51	< 0.001
Normalized BAS	0.760	≤ -0.38	79.81	64.34	76.92	68.15	73.60	2.24	0.31	< 0.001
Lym (%)	0.659	≤ 22.30	67.61	57.34	70.24	54.31	63.48	1.58	0.56	< 0.001
Monocytes (%)	0.694	> 6.50	71.36	62.94	74.15	59.60	67.98	1.93	0.46	< 0.001
EOS(%)	0.720	≤ 0.40	60.56	74.13	77.71	55.79	66.01	2.34	0.53	< 0.001
BAS(%)	0.736	≤ 0.10	56.34	79.72	80.54	55.08	65.73	2.78	0.55	< 0.001
NLR	0.652	> 3.99	52.58	72.73	74.17	50.73	60.67	1.93	0.65	< 0.001
Normalized PLR	0.696	> -0.13	52.11	85.31	84.09	54.46	65.45	3.55	0.56	< 0.001
**Combined variables**										
Lym + EOS	0.780	-	68.08	75.52	80.55	61.37	71.07	2.78	0.42	> 0.05^*^
Lym + BAS	0.802	-	76.53	71.33	79.90	67.11	74.44	2.67	0.33	< 0.05^*^
PLR + BAS	0.804	-	83.57	63.64	77.39	72.23	75.56	2.3	0.26	< 0.01^*^
EOS + BAS	0.787	-	84.98	60.84	76.37	73.12	75.28	2.17	0.25	0.013^#^
BAS% + MO%	0.805	-	79.34	71.33	80.48	69.86	76.12	2.77	0.29	< 0.01^*^
Lym + MO%	0.810	-	65.26	83.92	85.81	61.86	72.75	4.06	0.41	≤ 0.01*
BAS + MO%	0.810	-	84.51	67.13	79.29	74.42	77.53	2.57	0.23	< 0.01^*^

The cutoff values were selected by Youden Index

*Lym* Lymphocytes, *BAS* Basophils, *EOS* Eosinophils, *MO* Monocytes, *PLT* Platelets, *NLR* neutrophil-to-lymphocyte ratio, *PLR* platelet-to-lymphocyte ratio, *AUC* area under the curve, *PPV* positive predictive values, *NPV* negative predictive values, + *LR* positive likelihood ratios, *-LR* negative likelihood ratios

^*^compared with the AUC of each corresponding single variable

^#^compared with the AUC of BAS

The ROC analysis revealed that BAS, Lym, EOS counts, BAS (%), EOS (%), and PLR exhibited the highest AUCs for predicting the diagnosis of non-severe cases (0.760, 0.766, 0.748, 0.736, 0.720, 0.696, all *P* < 0.001) (Table 4). Logistic regressions with binary outcomes (Ct < 35) and two variables (normalized BAS, normalized Lym, normalized BAS, and normalized PLR) were performed. The logistic regression equations were as follows:

- ① $$\text{ln}\left(\frac{\text{P}\left(\text{Ct}<35\right)}{1-\text{P}\left(\text{Ct}<35\right)}\right)=0.424-0.876\times \text{normalized BAS}-0.720\times \text{normalized Lym}$$ 
- ② $$\ln\left(\frac{\mathrm P\left(\mathrm{Ct}<35\right)}{1-\mathrm P\left(\mathrm{Ct}<35\right)}\right)=0.544-1.055\times\mathrm{normalized}\;\mathrm{BAS}+0.860\times\mathrm{normalized}\;\mathrm{PLR}$$ 

Estimation results are presented in Tables 5 and 6.

**Table 5:** Variables estimation for logistic regression for the Basophils & Lymphocytes model

	Odds ratio	95% Confidence Interval		*P* value	Result
		Lower	Upper		
Intercept	1.528	-	-	0.001	Significant
Basophiles	0.417	0.291	0.596	< 0.001	Significant
Lymphocytes	0.487	0.353	0.672	< 0.001	Significant

**Table 6:** Variables estimation for logistic regression for the Basophils & PLR model

	Odds ratio	95% Confidence Interval		*P* value	Result
		Lower	Upper		
Intercept	1.723	-	-	< 0.001	Significant
Basophiles	0.348	0.249	0.488	< 0.001	Significant
PLR	2.362	1.488	3.751	< 0.001	Significant

**Fig. 2:**
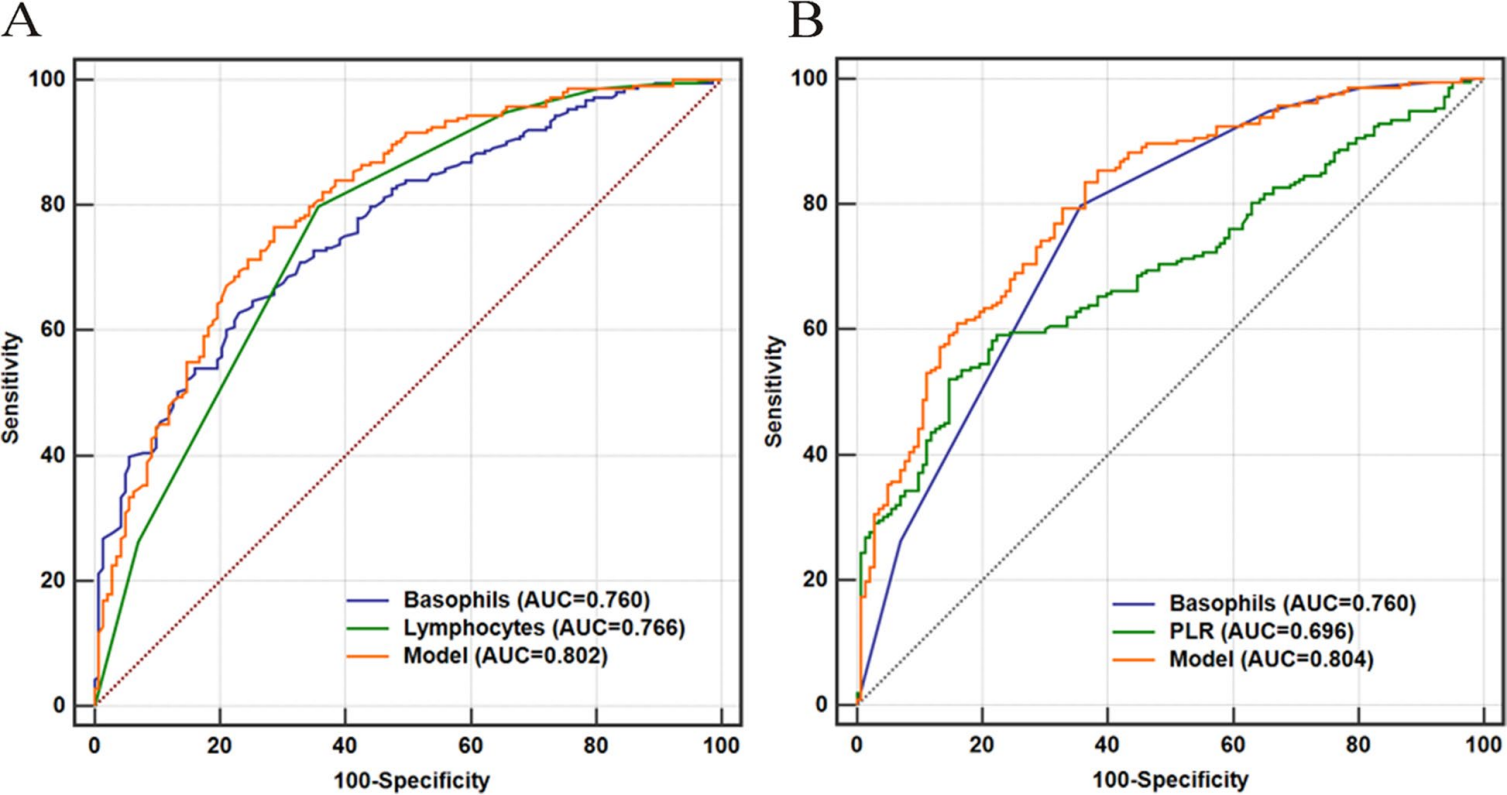
ROC curves for the model of basophils combined with lymphocytes and platelet-to-lymphocytes ratio (PLR) respectively in predicting positive Covid tests in patients with symptoms (**A**)&(**B**). **A** *n* = 356, AUC_model_ = 0.802 (95% CI, 0.757–0.843); AUC_basophils_ = 0.760 (95% CI, 0.713–0.804; *P* = 0.0075, compared with the model); AUC_lymphocytes_ = 0.766 (95% CI, 0.718–0.809; *P* = 0.8511 and = 0.0128, compared with basophils alone and the model respectively). **B** *n* = 356, AUC_model_ = 0.804 (95% CI, 0.759–0.844); AUC_basophils_ = 0.760 (95% CI, 0.713–0.804; *P* = 0.0036, compared with the model); AUC_PLR_ = 0.696 (95% CI, 0.645–0.743; *P* = 0.0527 and < 0.0001, compared with basophils alone and the model respectively)

A ROC analysis was performed using different combinations of these variables. The AUC for the combination of normalized BAS and normalized Lym was 0.802 (95% Confidence Interval [CI]: 0.757–0.843), which was significantly higher than that for normalized BAS (*P* = 0.0075) or normalized Lym alone (*P* = 0.0128) (Table 4, Fig. 2A). The AUC for the combination of normalized BAS and PLR was 0.804 (95% CI: 0.759–0.844), which was significantly higher than that for either normalized BAS (*P* = 0.0036) or normalized PLR alone (*P* < 0.0001) (Table 4, Fig. 2B).

### Optimal cut-off values for the non-severe cases prediction

The optimal cut-off values were calculated based on Youden’s index. The cut-off values for normalized BAS, normalized Lym, and normalized PLR were -0.38, -0.20, and -0.13, respectively. The corresponding values of BAS counts, Lym counts, and the PLR were 0.01 × 10^9^/L, 1.21 × 10^9^/L, 186.51, respectively.

### Predictive values of single and combined variables for asymptomatic carriers diagnosis

The ROC curve showed that the MO (%) provided the greatest AUC (0.708, *P* < 0.001) for predicting the diagnosis of asymptomatic carriers, whereas the AUC values for Lym and MO counts and the PLR were modestly predictive, ranging between 0.600 and 0.670 (Table 7). The optimal cut-off value of the MO (%) was 6.70%, which resulted in a sensitivity of 52.78% and a specificity of 84.13%.

**Table 7:** Predictive values of single and combined variables for positive Covid tests in asymptomatic patients (*n* = 171)

Characteristic variables	AUC	Cut off values^*^	Sensitivity(%)	Specificity(%)	PPV(%)	NPV(%)	Accuracy(%)	+ LR	-LR	Variable *P*
**Single variable**										
Lym	0.667	≤ 1.22	40.74	87.30	84.61	46.22	57.89	3.21	0.68	** < 0.001**
MO	0.601	> 0.51	36.11	82.54	78.00	42.97	53.22	2.07	0.77	** < 0.050**
MO (%)	0.708	> 6.70	52.78	84.13	85.08	50.96	64.33	3.33	0.56	** < 0.001**
PLR	0.627	> 183.85	37.96	85.71	82.00	44.62	55.56	2.66	0.72	** < 0.010**
**Combined variables**										
Lym + MO	0.687	> 0.6465	57.41	79.37	82.7	52.1	65.50	2.78	0.54	> 0.05^*^
PLR + MO	0.671	> 0.6190	53.70	74.60	78.40	48.50	61.40	2.11	0.62	> 0.05^*^
Lym + MO%	0.733	> 0.6742	57.41	87.30	85.6	54.5	68.42	4.52	0.49	> 0.05^*^
PLR + MO%	0.740	≤ 0.6738	57.41	85.71	87.3	54.0	67.84	4.02	0.50	** < 0.01**^**#**^

The cutoff values were selected by Youden Index

*Lym* Lymphocytes, *MO* Monocytes, *PLR* platelet-to-lymphocyte ratio, *AUC* area under the curve, *PPV* positive predictive values, *NPV* negative predictive values, + *LR* positive likelihood ratios, *-LR* negative likelihood ratios

^*^compared with the AUC of each corresponding single variable

^#^compared with the AUC of PLR

A combined application of the MO (%) and PLR for predicting positive COVID tests resulted in an AUC of 0.740, with a sensitivity and specificity of 57.41% and 85.71%, respectively.

### Comparisons of hematological parameters between the non-severe cases and asymptomatic carriers

Notably, non-severe cases exhibited lower PLT, Lym, EOS, and BAS counts, as well as lower Lym (%), EOS (%), and BAS (%) (all *P* < 0.001). Conversely, they exhibited higher MO counts, MO (%), Neu (%), NLR, PLR, and CRP levels (all *P* ≤ 0.01), compared with asymptomatic carriers (Table 8).

**Table 8:** Demographic data and hematological parameters comparisons between asymptomatic carriers and non-severe cases with positive results of nucleic acid testing

Characteristic variables	Asymptomatic carriers	Non-severe cases	*P* value
	n_1_ = 108	n_2_ = 213	
Male (n /%)^a^	50(46.30%)	111 (52.10%)	0.325
Age, Years^a^	34.00 (25.00, 49.75)	32.00 (27.00, 43.00)	0.610
Vaccination^c^	83 (76.85%)	185 (86.85%)	0.794
**Blood parameters**			
RBCs (3.68–5.13 × 10^12^/L)^a^	4.72 ( 4.36,5.21)	4.81 (4.45,5.21)	0.357
Haemoglobin (113–151 g/L)^b^	144.51 ± 16.08	144.85 ± 18.00	0.870
Haematocrit (33.5%-45.0%)^b^	43.17 ± 4.53	43.01 ± 4.75	0.773
Platelets (85–303 × 10^9^/L)^a^	222.00 (183.00,255.75)	190.00 (160.00,220.00)	** < 0.001**
WBCs (4.0–10.0 × 10^9^/L)^a^	6.19 (5.03,9.78)	6.15 (4.84,7.65)	0.696
Neutrophils (2.0–7.0 × 10^9^/L)^a^	4.24 (3.23,5.10)	4.41 (3.29,5.81)	0.197
Lymphocytes (0.8–4.0 × 10^9^/L)^a^	1.42 (0.98,1.91)	1.00 (0.61,1.48)	** < 0.001**
Eosinophils (0.02–0.5 × 10^9^/L)^a^	0.06 (0.02,0.11)	0.02 (0.01,0.06)	** < 0.001**
Monocytes (0.12–1 × 10^9^/L)^a^	0.43 (0.32,0.58)	0.51 (0.37,0.65)	**0.010**
Basophils (0.00–1.00 × 10^9^/L)^a^	0.01 (0.01,0.02)	0.01 (0.00,0.01)	** < 0.001**
Neutrophils (40–70%)^a^	67.10 (57.63,75.70)	72.90 (64.00,82.10)	** < 0.001**
Lym (20–40%)^a^	24.65 (16.50,31.28)	17.40 (10.20,25.90)	** < 0.001**
MO (3–10%)^a^	6.90 (5.03,9.78)	8.10 (6.25,11.40)	**0.004**
EOS(0.5–5%)^a^	1.05 (0.40,1.90)	0.30 (0.10,0.90)	** < 0.001**
BAS(0–1%)^a^	0.25 (0.10,0.30)	0.10 (0.10,0.20)	** < 0.001**
NLR^a^	2.73 (1.85,4.64)	4.22 (2.48,7.82)	** < 0.001**
PLR^a^	155.87 (120.71,224.47)	193.13(125.18,312.26)	**0.010**
C-reactive protein (0-10 mg/L)^a^	2.75 (1.20,7.68)	6.15 (3.08,12.85)	** < 0.001**

Bold font indicates statistical significance

*p* values comparing the group of COVID-19 cases and other groups are from Pearson’s χ^2^ test, Student’s *t* test (2-tailed) or Mann–Whitney U test

*Lym* Lymphocytes, *BAS* basophile, *EOS* eosinophils, *MO* Monocytes, *Neu* neutrophils, *NLR* neutrophil-to-lymphocyte ratio, *PLR* platelet -to-lymphocyteratio, *RBCs* red blood cells, *WBCs* white blood cells, *CRP* C-reactive protein

^a^median (IQR) values

^b^mean ± SD values

^c^Pearson Chi-Square; n1 = 92, n2 = 205

## Discussion

The Omicron variants of concern (VOCs) present properties of increased transmissibility and immune evasion, which were responsible for the recent pandemic in different regions worldwide. Although the risk of severe clinical outcomes from VOCs infection may be lower compared to prior variant infections, early identification and taking efficient measures to prevent its rapid transmission could play vital roles in preventing the overwhelming strain of medical and health systems in China [10]. This study describes the hematological characteristics of both symptomatic and asymptomatic patients infected with Omicron variants and assesses the value of BAS counts and other peripheral blood biomarkers, either independently or in combination, for differentiating infected cases among close contacts.

In the current study, 66.36% of Omicron variant-infected cases presented mild symptoms, and 33.64% of infected cases were asymptomatic, which is consistent with previous reports [2, 8, 29]. Among these infected cases, a small subset of symptomatic and asymptomatic patients exhibited typical mild changes on chest CT, including peripheral pulmonary plaques and interstitial lesions [17]. This differs from prior variant infections, where changes in chest CT were commonly observed [2, 17]. These results provide further evidence supporting the reduced likelihood of severe clinical outcomes and hospitalization among patients infected with the Omicron variants compared to those infected with prior variants [2, 11].

Since the breakout of COVID-19, several peripheral blood biomarkers have been used to predict the diagnosis and prognosis of COVID-19 pneumonia and distinguish COVID-19 from influenza, considering their simple, efficient, and economic properties in clinical practice [16–20, 23, 25]. Consistent with previous reports [17], our current study showed that 9.35% of the infected patients presented with leukopenia, and 26.79% exhibited lymphopenia, regardless of the symptoms. However, only 35.20% of the infected patients had a reduction in EOS counts, which was substantially lower than the 75% reported in previous patients with COVID-19 pneumonia [17]. Then, patients were divided into a symptomatic group and an asymptomatic group. Notably, our results showed that non-severe cases had lower Lym, EOS, BAS, and PLT counts, but higher MO counts, NLRs, and PLRs than suspected cases; however, no significant differences were observed regarding EOS and BAS counts between asymptomatic carriers and asymptomatic close contacts with negative results of COVID tests. Interestingly, when excluding common cases from the non-severe group, we attained similar results as mentioned above (data not shown). This may be due to the different immune statuses of symptomatic and asymptomatic patients [18]. The innate immune system may play a predominant role in asymptomatic carriers [30]. However, the adaptive immune system may overwhelm the systemic balance in mild cases [18]. These results indicate that peripheral blood biomarkers, especially EOS and BAS, may play important roles in predicting the diagnosis of Omicron variant infections in symptomatic patients.

BAS are involved in the pathogenesis of viral infections [23, 31–34]. Several studies have shown that the BAS count or percentage in patients with COVID-19 is significantly reduced compared to controls [16, 20, 22, 23, 35], and this trend extends to patients with severe COVID-19 when compared to those with mild or moderate COVID-19 [25–27]. Patients with COVID-19 show a tendency toward basopenia, suggesting that BAS plays a protective role against SARS-CoV-2 infection [18]. Rodriguez et al. found that BAS can promote an immunoglobulin (Ig) G response against SARS-CoV-2 because of its ability to secrete interleukin (IL)-4 [36]. IL-4 is an important inflammatory factor involved in enhancing B-cell activity against infection [31]. Our current study revealed a lower BAS score in patients with mild disease than in asymptomatic carriers. This trend aligns with previous studies, indicating that the lower the BAS count, the more severe the disease [27, 37]. A possible mechanism may involve the reduced expression of the prostaglandin D2 receptor, known as CRTH2, on the surface of BAS [18].

The mononuclear phagocyte (MNP) system, which includes MO and macrophages, plays an important role in COVID-19-related hyper-inflammation [38]. The proportion of MNPs in the bronchoalveolar fluid of COVID-19 patients has increased [39]. MO secreting IL-6 has also been detected in the peripheral blood of patients with COVID-19 in intensive care units [39]. Peripheral blood MO counts are helpful in differentiating influenza infection from COVID-19 infection [19]. However, no differences in peripheral blood MO counts were observed between patients with COVID-19 and healthy individuals in previous studies [17, 19]. Our study findings showed that peripheral blood MO counts and MO (%) were lower in patients with positive nucleic acid results in both symptomatic and asymptomatic groups. Considering the significant difference in WBC counts between patients with positive and negative nucleic acid results, the clinical significance of the changes in peripheral blood MO counts (%) was limited.

Our current study showed that Lym, BAS, and EOS counts made the most significant contribution to differentiating Omicron variant infections among close contacts with symptoms. The predictive value for the diagnosis of Omicron variants infection was significantly improved when the BAS count was combined with the Lym count or PLR (AUC_BAS+Lym_ vs. AUC_BAS_ vs. AUC _Lym_: 0.802 vs. 0.760 vs. 0.766; AUC_BAS+PLR_ vs. AUC_BAS_ vs. AUC_PLR_: 0.804 vs. 0.760 vs. 0.696). The BAS exhibited a sensitivity, specificity, NPV, PPV, and accuracy of 79.81%, 64.34%, 68.15%, 76.92%, and 73.60%, respectively. Compared with the corresponding values calculated from Lym alone, BAS combined with Lym demonstrated higher sensitivity (76.53% for the combination, 62.91% for Lym alone) and negative predictive value (67.11% for the combination, 58.2% for Lym alone). Additionally, the corresponding specificity (71.33% for the combination) and positive predictive value (79.90% for the combination) also improved when compared to using BAS alone. Similarly, the sensitivity (83.57% for the combination, 52.11% for PLR alone) and negative predictive value (72.23% for the combination, 54.46% for PLR alone, and 68.15% for BAS alone) improved when the BAS count was combined with the PLR. This indicates that the combined use of optimal cut-off values of BAS and Lym counts, or PLRs, contributed to an improved prediction of non-severe case diagnosis.

When excluding common cases from non-severe cases, we obtained similar results. Specifically, in the context of mild cases only, the predictive value for the diagnosis of Omicron variant infections was also significantly improved when the BAS count was combined with the Lym count or PLR (AUC_BAS+Lym_ vs. AUC_BAS_ vs. AUC_Lym_: 0.806 vs. 0.763 vs. 0.771, all *P* < 0.05; AUC_BAS+PLR_ vs. AUC_BAS_ vs. AUC_PLR_: 0.808 vs. 0.763 vs. 0.704, all *P* < 0.05). These findings suggest that BAS, along with Lym or PLR, primarily contributed to the improved prediction of mild case diagnosis. Unfortunately, the value of these hematological parameters for screening common cases of Omicron infections remains unknown due to the small population size, which was insufficient for analysis. Nonetheless, previous studies have provided evidence indicating that the combination of the EOS count and NLR can be used to diagnose COVID-19 pneumonia [17], or that the BAS (%) combined with the MO count could distinguish COVID-19 pneumonia from influenza infection [19].

For asymptomatic close contacts, the predictive value of combining the PLR with the MO (%) (AUC = 0.740) for positive COVID tests was notably improved compared with that of the PLR alone (AUC = 0.627, *P* < 0.01). However, the value of peripheral parameters for predicting the diagnosis of asymptomatic carriers is limited for the following reasons. Firstly, the diagnostic performance, as assessed by the AUC, of combining the PLR with MO (%) (AUC = 0.740) for identifying positive COVID tests was not significantly improved compared to relying solely on the MO (%) (AUC = 0.708, *P* > 0.05). Secondly, WBC counts were lower in positive cases than in negative cases *(P* = 0.001), which made the value of the MO (%) uncertain.

This study had certain limitations. First, peripheral blood parameters were not measured daily, and few patients were willing to undergo blood tests again when they felt better. Therefore, this study did not continuously monitor the changes in peripheral blood parameters. Furthermore, the relationship between the alterations in these parameters and disease prognosis remains unknown. Second, this study only included close contacts with negative serum influenza A and B IgM; therefore, whether this method can distinguish Omicron infection from influenza infection remains unclear. Third, the reason for the changes in the BAS count was not explored in this study. Further studies are needed to elucidate the mechanisms by which BAS modulate the immune response to Omicron variants. Nevertheless, this study provided a method for rapidly discerning Omicron variant infection among close contacts with negative serum influenza A and B IgM at an early stage, especially in the symptomatic group.

## Conclusions

Peripheral blood BAS counts, alone or in combination with other blood parameters, may serve as helpful, convenient, and efficient biomarkers for the diagnosis and assessment of symptomatic patients infected with Omicron variants. Circulating BAS counts lower than 0.01 $\times$ 10^9^/L may play a role in distinguishing patients with non-severe Omicron infection, with a significantly improved predictive value when combined with the PLR or Lym count for confirming the diagnosis. The peripheral blood BAS count test can be selected as an effective indicator because it is economical, simple, and rapid.

## Data Availability

Data is provided within the manuscript.The datasets used and/or analyzed during the current study are available from the corresponding author on reasonable request.

## Abbreviations

AUC: Area under the receiver operating characteristic curve
BAS: Basophils
CRP: C-reactive protein
CT: Computed tomography
COVID-19: Coronavirus disease 2019
Ct: Cycle threshold
EOS: Eosinophils
IgM: Immunoglobulin M
IL-4: Interleukin-4
IL-6: Interleukin-6
Lym: Lymphocytes
+ LR: Positive likelihood ratios
-LR: Negative likelihood ratios
MNP: Mononuclear phagocytic
MO: Monocytes
Neu: Neutrophils
NLR: Neutrophil-to-lymphocyte ratio
NPV: Negative predictive values
PLR: Platelet-to-lymphocyte ratio
RT-PCR: Reverse transcriptase-polymerase chain reaction
PPV: Positive predictive values
RBCs: Red blood cells
SARS-CoV-2: Severe acute respiratory syndrome coronavirus 2
VOCs: Variants of concern
WBC: White blood cell
